# Individual consistency in migration strategies of a tropical seabird, the Round Island petrel

**DOI:** 10.1186/s40462-022-00311-y

**Published:** 2022-03-14

**Authors:** Kirsty A. Franklin, Ken Norris, Jennifer A. Gill, Norman Ratcliffe, Anne-Sophie Bonnet-Lebrun, Simon J. Butler, Nik C. Cole, Carl G. Jones, Simeon Lisovski, Kevin Ruhomaun, Vikash Tatayah, Malcolm A. C. Nicoll

**Affiliations:** 1 School of Biological Sciences, University of East Anglia, Norwich Research Park, Norwich, UK; 2 Institute of Zoology, Zoological Society of London, Regent’s Park, London, UK; 3 Natural History Museum, Cromwell Road, London, UK; 4 British Antarctic Survey, High Cross, Madingley Road, Cambridge, UK; 5 Durrell Wildlife Conservation Trust, Les Augrès Manor, Trinity, Jersey, UK; 6 Mauritian Wildlife Foundation, Grannum Road, Vacoas, Mauritius; 7 Alfred Wegener Institute Helmholtz Center for Polar and Marine Research, Potsdam, Germany; 8 National Parks and Conservation Service (Government of Mauritius), Reduit, Mauritius

**Keywords:** Annual cycle, Geolocator, Flexibility, Individual variation, Non-breeding period, *Pterodroma*, Repeatability

## Abstract

**Background:**

In migratory species, the extent of within- and between-individual variation in migratory strategies can influence potential rates and directions of responses to environmental changes. Quantifying this variation requires tracking of many individuals on repeated migratory journeys. At temperate and higher latitudes, low levels of within-individual variation in migratory behaviours are common and may reflect repeated use of predictable resources in these seasonally-structured environments. However, variation in migratory behaviours in the tropics, where seasonal predictability of food resources can be weaker, remains largely unknown.

**Methods:**

Round Island petrels (*Pterodroma* sp.) are tropical, pelagic seabirds that breed all year round and perform long-distance migrations. Using multi-year geolocator tracking data from 62 individuals between 2009 and 2018, we quantify levels of within- and between-individual variation in non-breeding distributions and timings.

**Results:**

We found striking levels of between-individual variation in at-sea movements and timings, with non-breeding migrations to different areas occurring across much of the Indian Ocean and throughout the whole year. Despite this, repeat-tracking of individual petrels revealed remarkably high levels of spatial and temporal consistency in within-individual migratory behaviour, particularly for petrels that departed at similar times in different years and for those departing in the austral summer. However, while the same areas were used by individuals in different years, they were not necessarily used at the same times during the non-breeding period.

**Conclusions:**

Even in tropical systems with huge ranges of migratory routes and timings, our results suggest benefits of consistency in individual migratory behaviours. Identifying the factors that drive and maintain between-individual variation in migratory behaviour, and the consequences for breeding success and survival, will be key to understanding the consequences of environmental change across migratory ranges.

**Supplementary Information:**

The online version contains supplementary material available at 10.1186/s40462-022-00311-y.

## Background

Rapid changes in environmental conditions are affecting ecosystems, communities and species worldwide [58]. For migratory species that are dependent upon the availability of habitats, resources and conditions in multiple locations across migratory ranges, differing rates of change in these areas can greatly increase the potential for deleterious impacts at some point in the annual cycle [45]. Consequently, species lacking variability and flexibility in their migratory traits which might facilitate responses to changing environmental conditions could be at a disadvantage [18]. For example, among European breeding birds, species which have shown little or no phenological change in recent decades tend to also be those that are currently in population decline [38]. In order to identify potential constraints on migratory species’ responses to environmental change, we therefore need to understand variability and flexibility in migratory behaviour.

Recent advances in remote tracking technology have facilitated numerous studies following the movements of birds across multiple annual cycles [34]. These repeated measures of individuals allow investigation of spatiotemporal consistency (or, conversely, flexibility) in migration strategies within and among individuals (e.g., [8, 35]). As a result, there are now many avian studies which have investigated individual consistency in migratory timings (reviewed by Franklin et al., in review, [5]), and non-breeding locations [13, 42] and, more recently, year-to-year fidelity in migratory routes [54] and stopovers [8, 22]. Most studies have revealed high individual time- [17] and site-fidelity across years [19], with variation existing between individuals. However, some studies have also recorded individuals changing migratory behaviours between years [14, 35]. The occurrence and persistence of individual consistency in migratory behaviour may have emerged as a result of spatial and temporal predictability of resource availability [36], with familiarity of conditions at known locations and times being more beneficial than trying to locate optimal conditions at any given time [8, 60]. Predictable resource distributions might therefore be expected to result in low within-individual variation in space and time across years, and resource landscapes that are heterogeneous and predictable might be expected to result in between-individual variation and within-individual consistency [1]. By contrast, migratory species in environments in which resources are less predictable in space and time might be expected to show higher levels of within-individual flexibility. As most tracking studies have been conducted on species breeding at temperate and higher latitudes, levels of migratory consistency in less seasonal and unpredictable environments, such as tropical systems, remains unclear [10] (Franklin et al., in review).

Seabirds wintering in temperate and polar regions often associate with physical oceanographic features, such as oceanic fronts, shelf and ice edges or upwellings. These features, along with seasonal temperature and salinity gradients, tend to lead to temporally and spatially predictable prey aggregations [57]. Individual birds in these systems tend to have predictable migrations to one or more of these high-productivity ocean areas and show high levels of migration fidelity between years (e.g., [42]). By contrast, large areas of tropical oceans are often considered low in productivity and prey abundance, and have less marked seasonal variation in temperature, making prey aggregations unpredictable [59], but see [26]. Seabirds foraging at lower latitudes also often rely on subsurface marine predators, such as dolphin and tuna, that drive prey to the surface [3, 24, 49], and these events are likely to be less predictable than static oceanographic variables related to marine productivity. Non-breeding migrations of tropical species may therefore be less predictable both within- and between- individuals. However, only a very restricted number of studies have tracked individual tropical seabirds on multiple migrations [25, 44], and typically in such small sample sizes that quantifying within-individual variability has not yet been possible. So far for tropical seabirds, studies have mainly focused on variation in behaviours at breeding grounds, when individuals are constrained to a central location, and have revealed no consistent patterns in foraging site-fidelity [11, 40]. Consequently, it remains unclear whether consistent individual migratory behaviours occur in less predictable, tropical environments.

We addressed these questions using a tropical, pelagic seabird, the Round Island petrel, as a model species. This population of gadfly petrels (genus *Pterodroma*) breeds all-year round on Round Island, Mauritius, in the western Indian Ocean, and has been the focus of a long-term geolocator tracking project [39]. The mid-ocean location of Round Island means that petrel migrations could potentially occur in any compass direction and for a huge range of distances. Here we estimate how repeatable individual Round Island petrels are in their migratory timings (arrival to and departure from Round Island), and migration duration (time away from Round Island). We then use the earth mover’s distance (EMD), an algorithm originally developed for image comparison [46] and subsequently adapted to quantify similarity between spatial distributions [27], to quantify a) spatial consistency of petrel migrations across the entire non-breeding distribution, and b) whether individuals consistently occur in the same locations at the same stages (~ monthly) of their migration schedules.

## Methods

### Study site and species

This study was carried out at Round Island Nature Reserve (19.85° S, 57.78° E), a 219-ha island situated 22.5 km off the North coast of mainland Mauritius. The climate of Mauritius and the surrounding ocean is strongly seasonal and can be divided into two broad seasons: the austral winter (hereafter ‘winter’), and the austral summer (hereafter ‘summer’). The former typically runs from May to September and the latter from October to April, which reflects the influence of the monsoon circulation of the Indian Ocean [48]. Round Island is the only confirmed colony in the Indian Ocean of an unusual population of *Pterodroma* petrels, known as the Round Island petrel. Genetic evidence has shown this population to comprise at least three species of *Pterodroma* petrel (Trindade petrel, *P. arminjoniana*, Kermadec petrel, *P. neglecta* and Herald petrel, *P. heraldica*), which breed and extensively hybridise on the island [4, 6, 7, 52, 53]. Round Island petrels nest on the ground, typically under rock ledges or among piles of boulders. Eggs and chicks can be present in any month of the year, although there is a peak in egg-laying in August-October [53]. As petrel breeding activity is typically lowest on the island in May each year, we selected 1 June as the start of the petrel breeding calendar and numbered days sequentially from this origin [39].

### Geolocator deployment

Petrel surveys have been undertaken monthly since 2001, and involve regular visits to known nesting areas, ringing of adults and chicks (with South African Bird Ringing Unit numbered rings), and their subsequent recapture. Between 2009 and 2016, 421 light-level geolocators (GLS) were deployed on adult petrels. GLS were attached to the tarsus via a 1 or 0.75 mm thick Salbex (an industrial grade PVC; Sallu Plastics, Redditch, UK) colour-ring. Between 2009 and 2012, MK15 British Antarctic Survey geolocators (Cambridge, UK) were deployed, and during 2014 and 2016, Intigeo C250 and Intigeo C330 (Migrate Technology, Cambridge, UK) were deployed. The total device weight (including plastic ring) across all three tag types amounted to 3.6—3.9 g, which represents approximately 1.0% of the mean body mass of adult petrels (374 g). For the first three deployment periods, the tagged petrels were caught during targeted searches, whereas later GLS deployments occurred during the standard monthly petrel breeding surveys; these birds were predominantly resting on the island and not directly observed in a breeding attempt. Loggers were opportunistically recovered a minimum of one year later, during breeding surveys or during occasional specific searches. All loggers underwent a 3- to 5-day calibration period at a known location (Round Island, or mainland Mauritius (20.25° S, 57.44° E)) pre- and post- deployment. Details of the numbers of tags deployed and recovered until the end of December 2019 are provided in Additional File 1: Table S1.

### Geolocation data processing

At-sea locations for each individual were estimated from raw light-level data using the threshold method of estimating positions based on twilight events (i.e., sunrise and sunset transitions; [32]). Twilight events were defined using the preprocessLight function in the R package TwGeos [33] using a light intensity threshold of 4 and 1, for MK15 and Intigeo tags, respectively. The corresponding zenith angle was defined separately for each tag from the sunrise and sunset times recorded during the pre-deployment calibration period (range of 94.0° to 96.9° for MK15 tags and 96.8° to 99.0° for Intigeo tags). A Bayesian framework was used to refine the initial positions estimated from the threshold method and to derive uncertainty estimates. The R package SGAT [61] uses Markov Chain Monte Carlo (MCMC) simulations allowing the incorporation of: (1) a spatial probability mask, (2) sea-surface temperatures (SST) recorded by the tag in relation to global remote sensed SST maps, (3) prior definition of the error distribution of twilight events (twilight model) and (4) a flight speed distribution (behavioural model), to refine location estimates. The twilight model should reflect the expected error in detecting the real time of sunrise and sunset. Since the petrels spend a substantial amount of time sitting when at the breeding site (obscuring the light sensor) and can travel many hundreds of kilometres when still associated with the island, we could not use twilight times from a known location (i.e., Round Island) to parameterise the twilight model. We therefore used a rather conservative prior (log-normal distribution: meanlog = 2.2, sdlog = 1.0) describing a large variation in the discrepancy between the real and recorded twilight events. The spatial probability mask was constructed on the premise that birds only use marine environments when away from the colony (probability of 0 for positions on land). The probability at sea was further refined for each individual using remotely sensed sea-surface temperatures (weekly means on a 1.0° × 1.0° resolution downloaded from NOAA’s Optimum Interpolation Sea Surface Temperature V2 dataset: https://www.esrl.noaa.gov/psd/data/gridded/data.noaa.oisst.v2.html) and the SST values recorded by the loggers. The potential flight speeds were modelled following a gamma distribution (shape = 2.0, rate = 0.1). For each individual, we used these parameters and started by drawing an initial 2000 samples for burn-in and tuning of the proposal distribution using a modified model with relaxed assumptions. Then a further 300 samples were drawn three times to evaluate chain convergence before drawing another 4 MCMC chains of 3000 samples each to describe the posterior distribution. Tracks were summarised to produce median tracks and 95% credible intervals.

### Migratory timings

To identify the start and end dates of migration from GLS data, each day of GLS tracking was classified as one of two behavioural states (ashore on Round Island or at-sea) using a hidden Markov model (HMM). HMMs are a type of state‐space model, which decompose observed time‐series data into an observed sequence of discrete behavioural states. Behaviours were classified as either ashore or at-sea from the proportion of light interference during core daylight hours, the sum of daily wet records, and the distance from Round Island, using the R package depmixS4 [56]. These variables were chosen as when petrels are frequenting the colony, we expect; (1) a high degree of shading of the tag as individuals are sitting, (2) longer periods without immersion, and (3) petrels to remain close to the colony. Distance from colony (km) was derived from the SGAT processed median location estimates and calculated using the distGeo function in the R package geosphere [23], whereas light and immersion data were from the respective raw GLS files. All GLS models sampled light every minute and logged the maximum light recorded at 10-min intervals. To calculate light interference, we used the twilight times defined from the preprocessLight function to select out light recordings during daylight hours only. In contrast to the MK15 GLS, which record only low light levels, Intigeo GLS record the entire light range. Therefore, light samples with values of less than 64 or less than 100 were classed as interference for MK15 and Intigeo, respectively. Due to the different number of light samples that fall within each daylight period, this variable was calculated as the proportion of light samples with interference for each calendar day. GLS tested for saltwater immersion every 3 or every 30 s and stored the sum of positive tests at 10-min intervals, resulting in values between 0 (entirely dry), and 200 or 20 (entirely wet), for the MK15 and Intigeo GLS, respectively. The sum of these 144 values (number of 10-min periods in 24-h) were calculated for each calendar day. A Gaussian distribution was used to describe both distance from Round Island and immersion data, and a binomial distribution to describe light interference. Migration periods were defined as a sequence of consecutive days that were assigned to the same behaviour (at-sea) by the HMM for a period of at least three months, before switching to the other behavioural state (ashore). The duration of the migratory period was calculated using these dates.

### Spatial consistency

To investigate consistency in migratory locations within- and between- individuals, we used the median locations identified as the migration period from the HMM in combination with the earth mover’s distance (EMD; [27]) to create a matrix of space-use similarity estimates. In contrast to spatial overlap indices (where distributions with no spatial overlap have the same similarity value irrespective of their distance from one another), the EMD integrates a measure of spatial proximity in the similarity between different space-use distributions, by calculating the effort it takes to transform, for example, one migration track into another. EMD ‘effort’ values are therefore on a continuous scale, starting at zero for two identical migrations and increase with increasing dissimilarity. EMD was calculated for all petrel and year combinations, using Haversine distance with the ‘emd’ function in the R package move [28], using geographical coordinates directly as inputs.

In order to show how EMD relates to a widely used method for quantifying home range overlap, we also calculated relative overlap of petrel migrations using Bhattacharyya's affinity (BA; [16]). BA estimates range between 0 (no overlap) to 1 (identical distributions) and therefore do not quantify how dissimilar migratory distributions are if they do not overlap. For each individual and year, migratory locations were used to generate kernel utilisation distributions (UDs) using the R package adehabitatHR [9]. A fixed smoothing parameter (*h*) of 200 km was used to account for precision error around location estimates [41]. BA was calculated between all possible paired combinations of petrel UDs using the ‘kerneloverlap’ function in the R package adehabitatHR. This analysis was carried out in a Lambert azimuthal equal-area projection centred on the centroid of all positions. By comparing with BA, we were also able to investigate the impact of any scale-dependence on our analyses of EMD. As EMD is an absolute metric, individuals covering large migratory distances have greater capacity to differ and are thus more likely to generate large EMD values.

To investigate the spatiotemporal similarity of the same individual’s migrations in different years, two different approaches were used to define time: each petrel’s migrations was split into (1) 30-day *periods* irrespective of the migration start date (Additional File 1: Fig. S1a), meaning that a short period of time may be excluded for within-individual migrations of different durations; and into (2) six *stages* of equal duration (average of 29.4 days ± 4.7 SD; Additional File 1: Fig. S1b), meaning that the corresponding within-individual stage between migrations may be of a different length but in total spans the whole migratory period. EMD values were calculated for within-individual migrations for the same 30-day period/stage only (i.e., comparing the first 30-day period/stage of the first migration to the first 30-day period/stage of the second migration etc.), using the same method as described above.

### Statistical analysis

Repeatability (*R*) of (a) arrival to, (b) departure from Round Island and (c) migration duration were each estimated in a mixed-effects model framework, using 1000 bootstrap iterations to estimate confidence intervals (CIs), with the R package rptR [51]. Arrival and departure dates were recorded as days from 1 June; individuals with consecutive migrations spanning 1 June, (e.g., arriving on 30 May (day 364) in year one, and 2 June (day 2) in year two) had the first date converted to a negative day value to overcome the circular nature of this variable (the inclusion or exclusion of negative values had no significant impact on the estimated repeatability values). All three models were fitted with a Gaussian error family and included ‘individual identity’ as a random effect.

To investigate whether petrels arriving/departing in the different seasons differ in their levels of variability in migratory timings, the number of days between the earliest and latest date for each individual (i.e., within-individual variation) was calculated separately for arrival and departure dates, for petrels which consistently arrived or departed in either the summer or winter only. These values were included as the response variable in generalised linear models (GLMs) with gamma error family and log link function for arrival and departure dates separately, with season as a fixed effect.

To quantify spatial repeatability in individual petrel migrations and whether this varied with differences in departure timing, the EMD values comparing whole petrel migrations were included as the response variable in a GLM with the binary factor ‘same individual’ (0 as ‘no’ and 1 as ‘yes’), and the difference in days between the compared individuals’ departure dates from Round Island as fixed factors. An interaction between ‘same individual’ and difference in departure dates was also included. A significantly negative coefficient for the ‘same individual’ classification would indicate higher overlap (i.e., greater spatial similarity) within-individuals than between-individuals. The EMD values were continuous, non-negative and right-skewed; therefore, a gamma error family and identity link function were used. To examine if scale-dependence in EMD influenced these findings, we ran a second GLM with binomial error family and log link function with the same fixed factors, but this time with the BA values as the response variable.

To investigate within-individual consistency in distribution at different stages during the migration period, GLMs with gamma error family and identity link function were run with EMD values for each (a) stage or (b) period as the response variable, and difference in days between departure dates, and stage/period of migration (categorical variable including a level for whole migration) as fixed factors. An interaction between the two fixed factors was also included, to explore whether similar departure timing between years results in more consistent use of the same locations at the same time in each year. For all models, non-significant (p < 0.05) terms were sequentially removed using backwards stepwise deletion and significance of terms was determined using ANOVA. Pairwise comparisons among levels were calculated based on estimated marginal means and adjusted using post-hoc Tukey correction using the R package emmeans [31]. All analyses were conducted using R version 3.6.2 [47].

## Results

A total of 337 retrieved GLS loggers provided data on 267 complete migration tracks of 198 Round Island petrel individuals between 2009 and 2019 (Additional File 1: Table S1). This includes repeated tracking of the same individuals providing complete migration tracks during two (n = 57), three (n = 3) or four (n = 2) migratory periods. The five individuals with three or four migratory periods were a result of two separate non-consecutive GLS deployments, rather than birds evading capture for multiple years following deployment. These tracks provided arrival dates over a period of 2 to 4 years, and migration durations for up to 4 years, for 62 petrels. Due to logger failure during deployment, partial data recovery was possible for a number of GLS devices, providing a total of 76 petrels with between 2 and 5 departure dates from Round Island (Table 1).

**Table 1:** Repeatability estimates (*R*) from adult Round Island petrels with repeated tracks (2–5 years) for departure date from the breeding colony, arrival at the colony and duration of the migratory period

	N_ind_/N_rep_	*R*	Lower CI	Upper CI	p value
Departure date from RI	76/169	0.787	0.696	0.852	< 0.001
Arrival date at RI	62/131	0.813	0.715	0.883	< 0.001
Duration of migration	62/131	0.465	0.251	0.630	< 0.001

Given are the number of individuals (N_ind_), number of migratory tracks (N_rep_), lower and upper 95% CIs, and p values

### Timing of migration

**Fig. 1:**
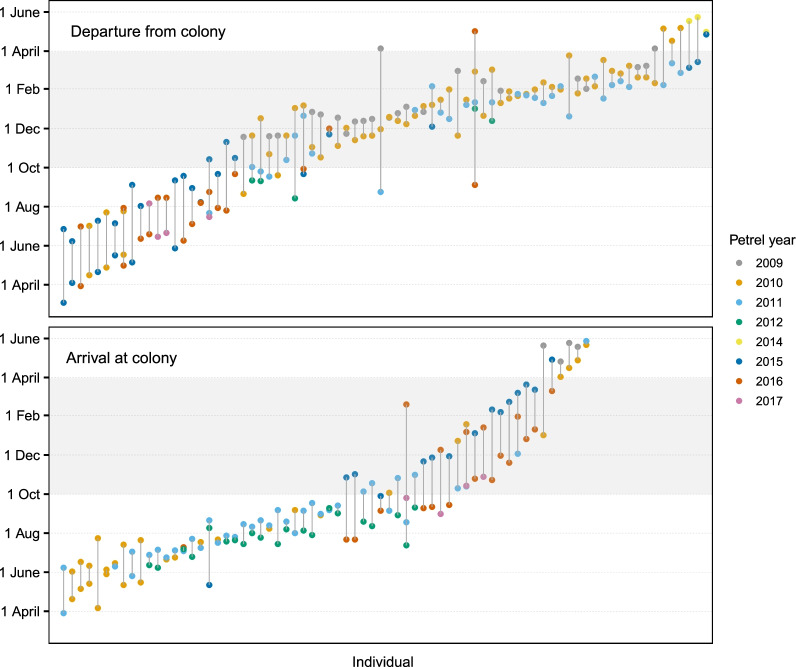
Dates of migration departure and arrival to/from the breeding colony (Round Island) of adult Round Island petrels tracked for more than one migration. Dates of the same individual are connected by vertical lines, and individuals are ordered from left to right by increasing mean date for departure and arrival, separately (therefore, individual one for departure is not the same as individual one for arrival). Filled circles are coloured by the petrel year (i.e., 2009 = 2009/2010) in which the departure or arrival took place. Grey shaded areas represent the austral summer

GLS tracking of sampled individuals covered departure and arrival dates of petrel migrations spanning all months. Repeated tracking of individual petrels indicated a much higher degree of consistency within- than between-individuals in all three migratory timings (arrival to, and departure from Round Island, and migration duration), with individual arrival dates to Round Island being the most repeatable (Fig. 1; Table 1). This repeatability was consistent across all years of the study period (Fig. 1). Although significantly repeatable, there is some variation within-individuals (median difference between latest and earliest departure date for each individual = 56 days (range 1–240 days) and for arrival = 47 days (range 2–220 days)), and individuals with a larger departure date range were also more likely to have a larger arrival date range (Pearson's correlation: r = 0.64, t = 6.38, df = 60, p < 0.0001). This within-individual repeatability in arrival and departure also differed between the two seasons which petrels arrived and departed, with the 17 birds departing in the winter (i.e. off-peak breeding period) having significantly higher within-individual variation than the 39 departing in the summer (i.e. peak breeding period, GLM: adjusted R^2^ = 0.25, β = 0.79, SE = 0.23, t = 6.55, p < 0.001), and the 10 birds arriving in the summer, having significantly higher within-individual variation than the 37 arriving in the winter (GLM: adjusted R^2^ = 0.48, β = 56.21, SE = 8.58, t = 6.55, p < 0.0001).

Migration durations of the 131 tracks (from the 62 repeatedly tracked petrels) lasted on average 175 days ± 28 SE (range 104–256 days). Despite a lower repeatability value for migration duration (Table 1), individuals showed relatively small mean individual ranges (difference between the largest and smallest record for each individual, in days; 22.8 ± 19.6 SD), highlighting that this low *R* value may reflect relatively low variance in migration duration among individuals. The variance components for each of the repeatability estimates can be found in Additional File 1: Table S8. 

### Spatial consistency in migratory journeys

**Fig. 2:**
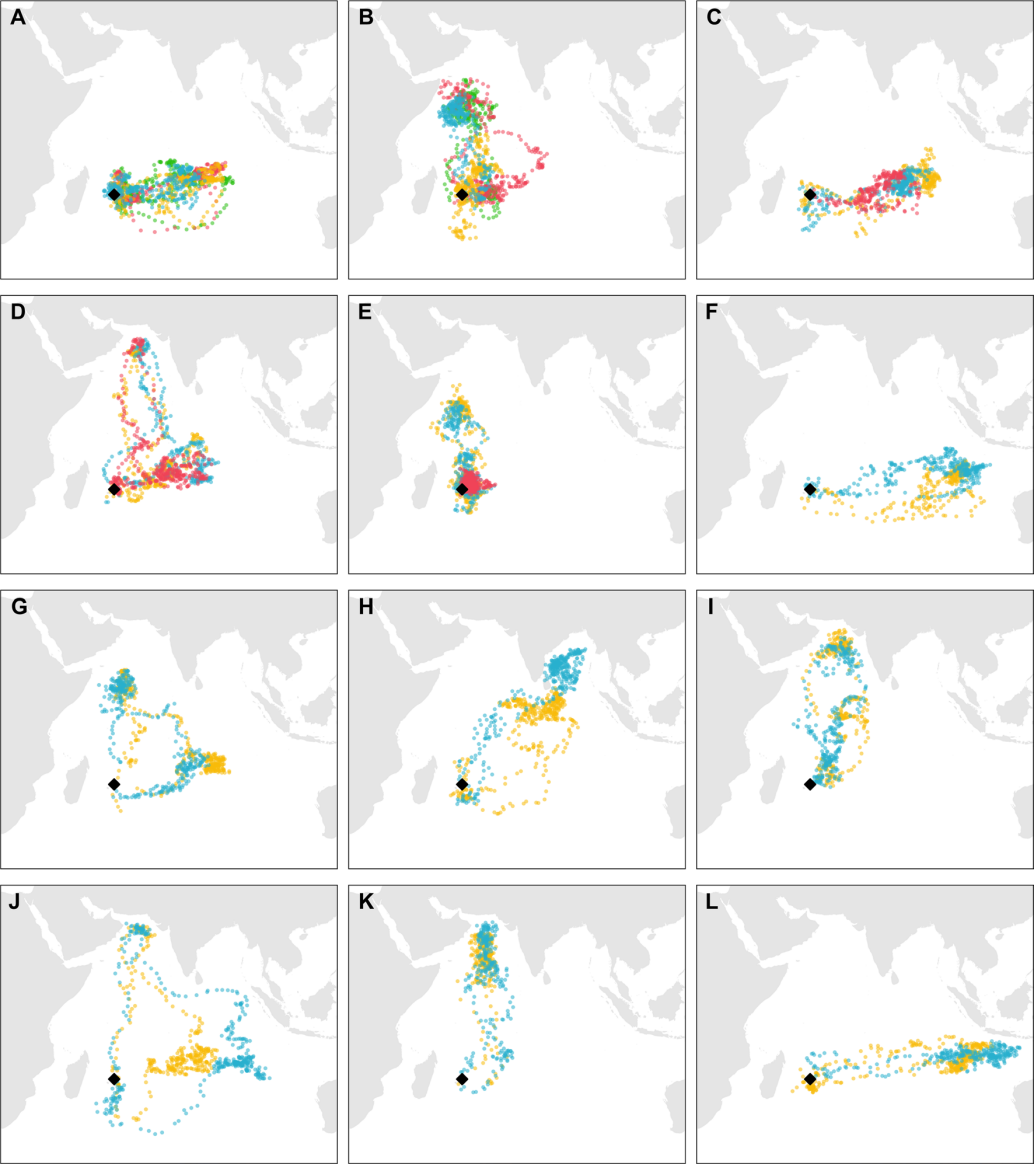
Example tracks of 12 individual Round Island petrels that have been tracked with geolocators over four (**A**, **B**), three (**C**–**E**), or two (**F**–**L**) migrations. Positions denote twice-daily median locations with different years illustrated in different colours. Black diamond indicates the location of Round Island, Mauritius. The tracking year that each set of colours represents can be found in Additional File 1: Table S7. Note that positions often overlap between years and hence might be partly obscured

**Fig. 3:**
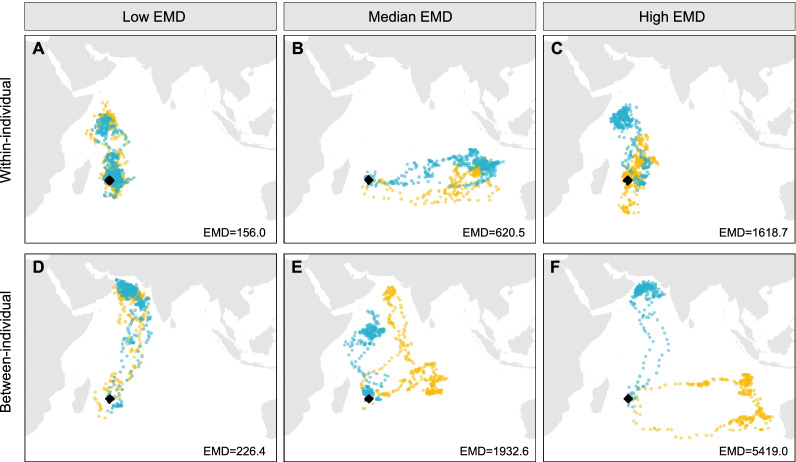
Example tracks of Round Island petrels with low (**A**, **D**), moderate (**B**, **E**), and high (**C**, **F**) within-individual (**A**–**C**) and between-individual (**D**–**F**) earth mover’s distance (EMD) migration comparisons. Moderate EMD values are based on the median values for within- and between-individual migration comparisons separately. Positions denote twice-daily median locations with the two different years illustrated in different colours. Black diamond indicates the location of Round Island, Mauritius. The tracking year that each set of colours represents can be found in Additional File 1: Table S7

**Fig. 4:**
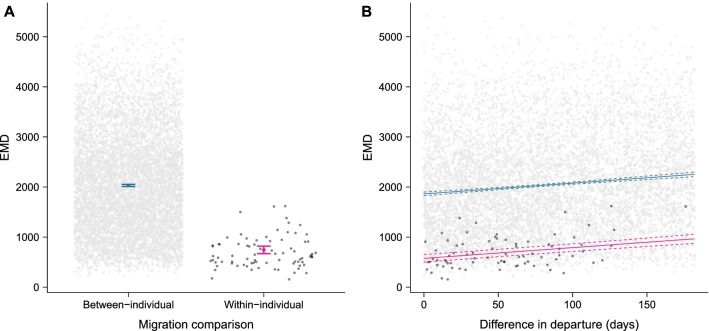
Predicted earth mover’s distance (EMD) values (lower values indicate more similar migrations) from a generalised linear model (GLM) of **A** between- and within-individual migration comparisons (error bars ± 95% confidence intervals), and **B** the difference in departure timing (fitted lines ± 95% confidence intervals). Pink (within-individual) and blue (between-individual) estimates are from GLM and raw data (filled circles) are shown for within- (black) and between-individual (grey) EMD values, separately. Model predictions for **A** are based on the median value for difference in departure of 79 days

Round Island petrels showed striking levels of between-individual variation in migratory journeys, with individuals undertaking non-breeding migrations to different areas across much of the Indian Ocean north of ~ 35°S (Fig. 2; Additional File 1: Fig. S2). Most birds migrated north to the Somali Basin and further into the Arabian Sea, or east across the central Indian Ocean between 10 and 20°S. Comparatively few travelled into the Bay of Bengal or Western Australian Basin, but all petrels largely avoided nearshore/shelf waters (Fig. 2; Additional File 1: Fig. S2). Despite this large between-individual spatial variation, repeated tracking of individual petrels for up to 4 years indicates remarkably high levels of spatial consistency (Figs. 2, 3), with within-individual petrel migrations being significantly more similar than between-individual migrations (Figs. 3, 4a; Table 2; Additional File 1: Table S2). EMD values for within-individual petrel migration comparisons ranged from 156.0 to 1618.7 compared to a range of 226.4 to 5419.0 for between-individual migrations (Figs. 3, 4a). The highest EMD value of 5419.0 indicates, in this case, the difference between individuals travelling north to the Arabian Sea versus east to the Western Australian Basin (Fig. 3f). The relative overlap of this pair of migrations when calculated using BA revealed a very low overlap of 0.06 (Additional File 1: Fig. S3a). However, as BA does not take into account the spatial distance between migrations, the same BA value can be observed across a range of EMD values, which represent paired migrations that are relatively close together in space (EMD = 2090.8; Additional File 1: Fig. S3c) or far apart (EMD = 5419.0; Additional File 1: Fig. S3a).

**Table 2:** Results of generalised linear models to investigate (a) the similarity of petrel whole migrations within- and between-individuals and (b) the spatiotemporal similarity of within-individual petrel migrations when split into six equal stages, using earth mover’s distance (EMD) ‘effort’ values

Variable	Estimate ± SE	*t* value	p value
(a) Whole migration EMD comparisons (R^2^ = 0.020)			
(Intercept)	1862.17 ± 19.17	97.14	** < 0.001**
1 (Same individual)	− 1287.08 ± 39.38	− 32.69	** < 0.001**
Difference in departure	2.15 ± 0.20	10.60	** < 0.001**
(b) Six stage EMD comparisons (R^2^ = 0.061)			
(Intercept)	500.51 ± 62.80	8.0	** < 0.001**
Stage*			
Stage 1	234.33 ± 90.04	2.6	**0.01**
Stage 2	359.11 ± 98.47	3.6	** < 0.001**
Stage 3	398.67 ± 101.20	3.9	** < 0.001**
Stage 4	355.13 ± 98.20	3.6	** < 0.001**
Stage 5	448.60 ± 104.67	4.3	** < 0.001**
Stage 6	543.29 ± 111.35	4.9	** < 0.001**
Difference in departure	3.94 ± 0.84	4.7	** < 0.001**

Minimum adequate models are shown and categorical variables are being compared to reference levels; for the ‘same individual’ binary variable, this is 0 (different individuals), and for ‘stage’, this is the whole migration. Significant effects (p < 0.05) are highlighted in bold

*The full pairwise comparisons for the categorical variable ‘stage’ can be found in Table S4

Within-individual spatial consistency was also significantly higher for individuals that departed Round Island at more similar times in each year (Fig. 4b; Table 2; Additional File 1: Table S2). However, this effect is very small in comparison to the large amount of variation in EMD values across the range of differences in departure timing (R^2^ = 0.020; Fig. 4). There was no significant interaction between ‘same individual’ and difference in departure dates meaning that the greater similarity of migratory ranges for birds departing at similar times applies both within- and between- individuals (Additional File 1: Table S2). Overall, comparison with the BA analysis showed both methods yielded broadly consistent results suggesting any scale-dependence in EMD values does not vary systematically in relation to difference in departure date, and thus does not affect our conclusions (Additional File 1: Tables S2, S3).

**Fig. 5:**
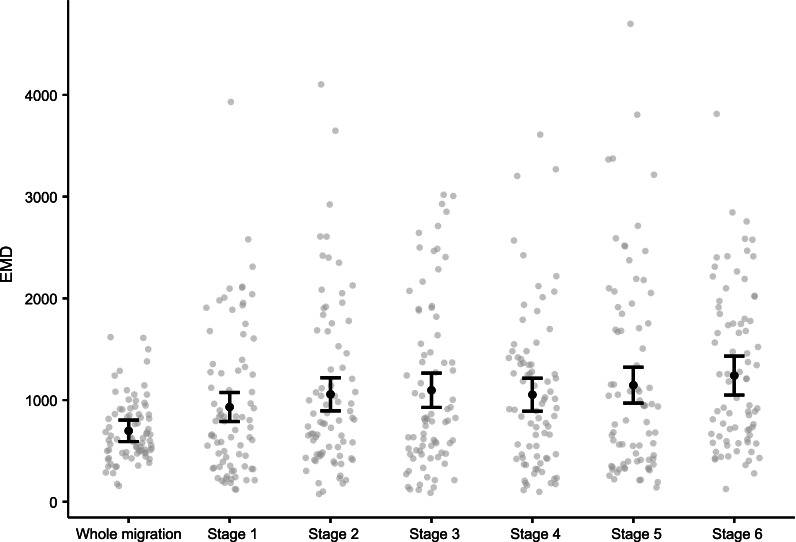
Predicted earth mover’s distance (EMD) values from generalised linear model (error bars ± 95% confidence intervals) and raw data (grey circles) of within-individual migration comparisons, for whole migration comparisons, and migrations when divided into six stages of equal size. Model predictions are based on the median value (50 days) for difference in departure

Despite high within-individual consistency in entire migratory journeys, individual Round Island petrels do not always use the same at-sea locations at the same stage or period within their migratory journeys. EMD values calculated for each stage/period of each petrel’s migrations were slightly, but significantly, higher than the EMD values for the whole migration (Fig. 5; Table 2; Additional File 1: Tables S2, S4–S6). These differences in the EMD values across stages/periods are not a result of individuals departing later or earlier in one migration compared to the other, as there was no significant interaction between stage/period and the difference in departure dates between years. Nonetheless, the overall pattern of petrels that departed Round Island at more similar times of year having more similar migrations still exists (Table 2).

## Discussion

Tropical, migratory Round Island petrels have striking levels of between-individual variation in at-sea movement patterns, with individuals undertaking migrations across much of the Indian Ocean and throughout the whole year. However, repeat tracking of individuals across the non-breeding period revealed very little within-individual variation, with migratory journeys being remarkably consistent in both space and time.

The areas frequented by Round Island petrels during migration cover much of the Indian Ocean north of ~ 35°S. Compared with most other studies tracking seabirds breeding in the western Indian Ocean, albeit in smaller numbers, the range of areas used by Round Island petrels during the non-breeding period is particularly large [30, 44]. GLS tracking of sooty terns (*Onychoprion fuscatus*) from Bird Island, Seychelles, also revealed use of a range of different non-breeding areas, three of which (the Bay of Bengal, northeast to an area straddling the Chagos-Laccadive plateau, southeast to an area on each side of the 90 East Ridge; [25]) are also used by Round Island petrels. However, the majority of Indian Ocean seabirds for which migratory journeys have been tracked, including the closely related Barau’s petrel (*Pterodroma baraui*), which nests on nearby Réunion Island, tend to show consistent eastward migrations to specific areas of the central and eastern Indian Ocean [44]. Systems with large non-breeding distributions, as seen in the Round Island petrel system, therefore provide ideal opportunities to explore the degree of within- and between-individual variation in migratory behaviour.

Although migration strategies were highly variable across the population, individuals used distinct areas within the overall range in a consistent manner across years. Individual consistency in space use has been shown for many migratory bird species, from great reed warblers (*Acrocephalus arundinaceus*) to Atlantic puffins (*Fratercula arctica*) [20, 22], however, this topic has received very little attention for migratory birds breeding in the tropics (e.g., [25]). Our results suggest that familiarity with locations may be more beneficial than tracking current environmental conditions even when resources may be patchy and unpredictable, such as those in tropical systems [2, 59]. Further, the fact that individuals which depart closer together in time are likely to have more similar non-breeding distributions may suggest that the meteorological or oceanographic conditions at departure can influence individual non-breeding distributions (but see [12]). However, we also observed temporal variation in space use, with EMD values being higher (i.e., migrations were less similar) when split into stages/periods, compared to the migration as a whole. This suggests a degree of temporal flexibility, such that individuals use the same areas in different years, but not necessarily at the same time during the non-breeding period, which has also been shown for other seabirds [37, 54]. This temporal flexibility therefore seems to only occur within the range of known areas for a particular individual, suggesting that relying on familiar areas is more beneficial than switching to a new location [36], and implies that temporal variation in resource availability may not be very large at these scales. Additionally, as gadfly petrel flying behaviour is strongly affected by wind conditions [50, 55] and thus how fast petrels move through their environment may vary with the environmental conditions that they experience in each year. The EMD metric provided an effective method for measuring spatial dissimilarity in non-overlapping distributions. This is particularly valuable for systems with large, non-overlapping variation in the possible range of individual migratory distributions, such as Round Island petrels. This framework could easily be applied to other species to compare space-use patterns within and across taxa (although effects of scale-dependence may need to be considered).

Repeated tracking of individual petrels also indicated a high degree of consistency in migratory timings. This has been reported for many other species (mean ICC = 0.408 (95% confidence interval: 0.3–0.5; reviewed by Franklin et al., in review) suggesting consistent individual differences in migration phenology to be a common feature of migratory systems. Recently, Trindade petrels breeding on Trindade Island in the South Atlantic Ocean have also shown to maintain their breeding schedules year-to-year [29]. For tropical seabirds, breeding phenology can range from seasonal and synchronised breeders (e.g., Barau’s petrel; [43]), to aseasonal breeders, albeit in varying numbers across the year (e.g., Round Island petrels; [53]). Consequently, it is important to note that repeatability of individual phenologies may be naturally inflated when a large number of viable phenologies exist in a population. Despite this, Round Island petrels were still remarkably repeatable in their migratory timings with much lower within- than between-individual variation. While the exact breeding status and/or outcome of individual petrels on Round Island is rarely known, other studies on seabirds have shown that failed breeders and non-breeders may depart earlier from their colony in comparison to successful breeders [42, 62]. Regardless of this, we still found high repeatability in migratory departure (although not as high as arrival) without accounting for breeding outcome (i.e., success, failure, or if breeding was attempted). Calculating repeatability in migratory timings requires consistent methods of classifying phenological events. In our study, we used HMMs in order to assign dates of arrival and departure from Round Island in an objective and reproducible manner, which was particularly important given that the low spatial resolution available from GLS tracks can make identification of departure times from location data alone problematic.

While most individuals seem to follow a consistent migratory schedule, the differing levels of within-individual variation between the seasons suggests an influence of prevailing environmental conditions on timings of departure and arrival. Petrels arriving at Round Island in the winter (which show more consistent timings of arrival) are likely to breed during the peak breeding period, and could thus experience greater competition for resources, including nest sites, than birds arriving during the summer period (which are less consistent in arrival times). Arriving at a consistent time each year may facilitate synchronous mate arrival [21], which may be particularly important if pairs are to compete for nest sites. Although the petrels nest in a range of conditions across the island, most nests occur within a relatively small number of colonies. Nesting sites within these colonies are likely to be in high demand, particularly during the peak breeding period, when broken eggs with peck marks and young chicks with head wounds are often observed [53]. Observations from camera traps have also shown intra- and inter- specific fights at petrel nest sites (Franklin, pers. obs.). As the Round Island petrel is a hybrid species complex [4, 6, 7], it means that the population comprises individuals with a great deal of genetic variation dictated by evolutionary histories. This, together with the fact that petrels have an asynchronous breeding period, means that interactions with different environmental conditions (such as the semi-annual wind reversals as a result of the two monsoon periods in the Indian Ocean [48]), may have given rise to the diverse range of migration patterns. However, it is not yet clear whether there is any temporal structuring in the genotypes of petrels on Round Island (i.e., are certain petrel hybrids on the island at certain times of year), or if different genotypes have different migratory distributions, which may contribute to individual phenological and spatial variation.

## Conclusions

The small amount of within-individual variation suggests that consistency in migratory behaviours is favoured even in comparatively patchy and unpredictable tropical systems [59]. This consistency, together with the fact that birds can be found breeding on Round Island all year round, means that different individuals are potentially exposed to different environmental conditions and human-associated impacts, with potentially important consequences for breeding success (e.g., [15]), survival and, ultimately, the status of this population. The Round Island petrel population appears to have arisen relatively recently in time through range expansions of different *Pterodroma* taxa [4, 6, 7]. The high level of individual migratory consistency means that future changes in non-breeding distributions and timings will most likely reflect changes in the numbers of individuals undertaking different journeys. Determining what is driving the large levels of between-individual variation in this system will be key in revealing the implications of individual consistency for population demography, and the potential consequences of future environmental changes across the migratory range.

## Supplementary Information

**Additional file 1. Table S1**: Details of all geolocator deployments and recoveries on adult Round Island petrels from 2009 to 2019, and number of complete migrations which took place in each petrel year for the 62 petrels with repeat migrations. **Table S2**: Results of ANOVA tests for generalised linear model selection for the similarity of petrel migrations within- and between- individuals, using a) the earth mover’s distance (EMD) ‘effort’ values, and b) Bhattacharyya’s affinity (BA), and the spatiotemporal similarity of within-individual petrel migrations when split into c) six equal size stages, and d) 30-day periods, both using EMD. Significant effects (p < 0.05) are highlighted in bold. **Table S3**: Results of generalised linear model to investigate the similarity of petrel whole migrations within- and between- individuals, using Bhattacharyya's affinity (BA) values. Minimum adequate model is shown. Note, the binary categorical variable ‘same individual’ is being compared to the reference level of that variable, which is 0 (different individuals). Significant effects (p < 0.05) are highlighted in bold. **Table S4**: Pairwise comparisons between each level of the categorical variable ‘stage’ from the generalised linear model examining the spatiotemporal similarity of petrel migrations when split into six equal size stages, using earth mover’s distance (EMD) ‘effort’ values. Significant effects (p < 0.05) are highlighted in bold. **Table S5**: Results of generalised linear model to investigate the spatiotemporal similarity of within-individual petrel migrations when split into 30-day periods, using earth mover’s distance (EMD) ‘effort’ values. Minimum adequate model is shown and categorical variable ‘period’ is being compared to reference level of whole migration. Significant effects (p < 0.05) are highlighted in bold. **Table S6**: Pairwise comparisons between levels of the categorical variable ‘30-day period’ from the generalised linear model examining the spatiotemporal similarity of petrel migrations when split into 30-day periods, using earth mover’s distance (EMD) ‘effort’ values. Significant effects (p < 0.05) are highlighted in bold. **Table S7**: Colour of geographic coordinates in figures (main text and Additional File 1) and the corresponding year/s of each migration. **Table S8. **: The variance components and repeatability estimates (*R*) from adult Round Island petrels with repeated tracks (2-5 years) for departure date from the breeding colony, arrival at the colony and duration of the migratory period. **Figure S1**: Earth mover’s distance (EMD) values are calculated for both A) consecutive 30-day periods, irrespective of start date, and B) by splitting the migration into six equal stages. For A), this typically means that a short period of longer within-individual migrations is not included, however, there was evidence of individual consistency in migration duration and so this often only comprises a small proportion of the whole migration. Whereas for B), if one migration is longer than the other then each stage will comprise of more days than the other corresponding stage. **Figure S2**: Geographic coordinates (a) and tracks (b) from 62 adult Round Island petrels that have been tracked over multiple complete migrations (n = 131) with geolocators. Colours of lines and points represent the year of tracking (2009-17). Black diamond indicates the location of Round Island, Mauritius. **Figure S3.** Example tracks of between-individual Round Island petrel migration comparisons with the same relative overlap value (Bhattacharyya's affinity (BA) = 0.06), but with comparatively high (A), moderate (B), and low (C) earth mover’s distance ‘effort’ values. Positions denote twice-daily median locations with the two different years illustrated in different colours. Black diamond indicates the location of Round Island, Mauritius. The tracking year that each set of colours represents can be found in Additional File 1: Table S7.

## Data Availability

Tracking data are deposited on the Seabird Tracking database (www.seabirdtracking.org) handled by BirdLife International (ID: 1810). R scripts to reproduce analysis can be found on Github (https://github.com/kirstyfranklin/RIpetrel-consistency).

## Abbreviations

BA: Bhattacharyya's affinity
EMD: Earth mover’s distance
GLM: Generalised linear model
GLS: Geolocator
HMM: Hidden Markov model
MCMC: Markov Chain Monte Carlo
SST: Sea surface temperature
UD: Utilization distribution
