# Correction to: Individual consistency in migration strategies of a tropical seabird, the Round Island petrel

**DOI:** 10.1186/s40462-022-00317-6

**Published:** 2022-03-24

**Authors:** Kirsty A. Franklin, Ken Norris, Jennifer A. Gill, Norman Ratcliffe, Anne-Sophie Bonnet-Lebrun, Simon J. Butler, Nik C. Cole, Carl G. Jones, Simeon Lisovski, Kevin Ruhomaun, Vikash Tatayah, Malcolm A. C. Nicoll

**Affiliations:** 1 School of Biological Sciences, University of East Anglia, Norwich Research Park, Norwich, UK; 2 Institute of Zoology, Zoological Society of London, Regent’s Park, London, UK; 3 Natural History Museum, Cromwell Road, London, UK; 4 British Antarctic Survey, High Cross, Madingley Road, Cambridge, UK; 5 Durrell Wildlife Conservation Trust, Les Augrès Manor, Trinity, Jersey, UK; 6 Mauritian Wildlife Foundation, Grannum Road, Vacoas, Mauritius; 7 Alfred Wegener Institute Helmholtz Center for Polar and Marine Research, Potsdam, Germany; 8 National Parks and Conservation Service (Government of Mauritius), Reduit, Mauritius

## Correction to: Franklin *et al. Movement Ecology (2022) 10:13*https://doi.org/10.1186/s40462-022-00311-y

The original publication of this article was published with an old version of Additional file 1 due to an error in the publication process. This old version did not contain Additional file 1: Table S8. The updated additional file is available via this correction article, the original article has also been updated. The publisher apologizes to the authors and readers for the inconvenience.

## Supplementary Information

**Additional file 1**. Updated version of Additional file 1.

