# Supplementary material for: Correction to: Individual consistency in migration strategies of a tropical seabird, the Round Island petrel
Source: Mov Ecol. 2022 Mar 24;10:15. doi: 10.1186/s40462-022-00317-6 (PMC8953334; doi:10.1186/s40462-022-00317-6)
Supplement: Supplementary file 1 — Additional file 1. Updated version of Additional file 1. [file 40462_2022_317_MOESM1_ESM.docx]

**Additional file 1: Table S1.** Details of all geolocator deployments and recoveries on adult Round Island petrels from 2009-2019, and number of complete migrations which took place in each petrel year for the 62 petrels with repeat migrations.

|  |  |  | **Petrel year which migration took place** | | | | | | |
| --- | --- | --- | --- | --- | --- | --- | --- | --- | --- |
| **Petrel year** | **Number deployed** | **Number recovered*** | **2009** | **2010** | **2011** | **2014** | **2015** | **2016** | **2017** |
| 2009 | 135 | 110 | 20 | 24 |  |  |  |  |  |
| 2010 | 84 | 79 |  | 24 | 25 |  |  |  |  |
| 2011 | 28 | 18 |  |  |  |  |  |  |  |
| 2012 | 79 | 64 |  |  |  |  |  |  |  |
| 2014 | 49 | 36 |  |  |  | 3 | 20 | 6 |  |
| 2015 | 21 | 15 |  |  |  |  | 1 | 5 | 1 |
| 2016 | 25 | 15 |  |  |  |  |  | 2 |  |
| **Total** | **421** | **337** | **20** | **48** | **25** | **3** | **21** | **13** | **1** |

* Geolocators recovered between October 2010 and December 2019.

**Additional file 1: Table S2.** Results of ANOVA tests for generalised linear model selection for the similarity of petrel migrations within- and between- individuals, using a) the earth mover’s distance (EMD) ‘effort’ values, and b) Bhattacharyya's affinity (BA), and the spatiotemporal similarity of within-individual petrel migrations when split into c) six equal size stages, and d) 30-day periods, both using EMD. Significant effects (p < 0.05) are highlighted in bold.

| Model | df | Deviance | F value | P value |
| --- | --- | --- | --- | --- |
| a. Whole migration EMD comparisons |  |  |  |  |
| EMD ~ Same individual*Difference in departure vs. EMD ~ Same individual + Difference in departure | 1 | 0.096 | 0.409 | 0.522 |
| EMD ~ Same individual vs.  EMD ~ Same individual + Difference in departure | 1 | 26.728 | 113.11 | **< 0.001** |
| EMD ~ Difference in departure vs.  EMD ~ Same individual + Difference in departure | 1 | 62.679 | 265.24 | **< 0.001** |
| b. BA comparisons |  |  |  |  |
| BA ~ Same individual*Difference in departure vs.  BA ~ Same individual + Difference in departure | 1 | 0.727 | 0.73 | 0.394 |
| BA ~ Same individual vs.  BA ~ Same individual + Difference in departure | 1 | 22.316 | 22.32 | **< 0.001** |
| BA ~ Difference in departure vs.  BA ~ Same individual + Difference in departure | 1 | 41.415 | 41.42 | **< 0.001** |
| c. Six stage EMD comparisons |  |  |  |  |
| EMD ~ Stage*Difference in departure vs.  EMD ~ Stage + Difference in departure | 6 | 2.310 | 0.779 | 0.587 |
| EMD ~ Difference in departure vs.  EMD ~ Stage + Difference in departure | 6 | 15.975 | 5.425 | **< 0.001** |
| EMD ~ Stage vs.  EMD ~ Stage + Difference in departure | 1 | 11.186 | 22.792 | **< 0.001** |
| d. 30-day period EMD comparisons |  |  |  |  |
| EMD ~ Period*Difference in departure vs.  EMD ~ Period + Difference in departure | 7 | 4.744 | 1.43 | **0.190** |
| EMD ~ Period vs.  EMD ~ Period + Difference in departure | 1 | 11.977 | 25.23 | **< 0.001** |
| EMD ~ Difference in departure vs.  EMD ~ Period + Difference in departure | 7 | 16.362 | 4.92 | **< 0.001** |

**Additional file 1: Table S3.** Results of generalised linear model to investigate the similarity of petrel whole migrations within- and between- individuals, using Bhattacharyya's affinity (BA) values. Minimum adequate model is shown. Note, the binary categorical variable ‘same individual’ is being compared to the reference level of that variable, which is 0 (different individuals). Significant effects (p < 0.05) are highlighted in bold.

| Variable | Estimate ± SE | z value | P value |
| --- | --- | --- | --- |
| Whole migration BA comparisons (R^2^ = 0.007) | | | |
| (Intercept)  1 (Same individual)  Difference in departure | -0.829 ± 0.024  0.634 ± 0.063  -0.001 ± 0.000 | -34.355  10.110  -4.787 | **<0.001**  **<0.001**  **<0.001** |

**Additional file 1: Table S4.** Pairwise comparisons between each level of the categorical variable ‘stage’ from the generalised linear model examining the spatiotemporal similarity of petrel migrations when split into six equal size stages, using earth mover’s distance (EMD) ‘effort’ values. Significant effects (p < 0.05) are highlighted in bold.

| Contrast | Estimate ± SE | P value |
| --- | --- | --- |
| Whole migration – Stage 1  Whole migration – Stage 2  Whole migration – Stage 3  Whole migration – Stage 4  Whole migration – Stage 5  Whole migration – Stage 6  Stage 1 – Stage 2  Stage 1 – Stage 3  Stage 1 – Stage 4  Stage 1 – Stage 5  Stage 1 – Stage 6  Stage 2 – Stage 3  Stage 2 – Stage 4  Stage 2 – Stage 5  Stage 2 – Stage 6  Stage 3 – Stage 4  Stage 3 – Stage 5  Stage 3 – Stage 6  Stage 4 – Stage 5  Stage 4 – Stage 6  Stage 5 – Stage 6 | -243.3 ± 90.0  -359.1 ± 98.5  -398.7 ± 101.2  -355.1 ± 98.2  -448.6 ± 104.7  -543.3 ± 111.4  -124.8 ± 110.1  -164.3 ± 112.5  -120.8 ± 109.9  -214.3 ± 115.7  -309.0 ± 121.7  -39.6 ± 119.4  4.0 ± 116.8  -89.5 ± 122.3  -184.2 ± 128.1  43.5 ± 119.1  -49.9 ± 124.5  -144.6 ± 130.2  -93.5 ± 122.1  -188.2 ± 127.8  -94.7 ± 132.9 | 0.1251  **0.0049**  **0.0016**  **0.0055**  **0.0004**  **< 0.0001**  0.9180  0.7685  0.9285  0.5123  0.1458  0.9999  1.0000  0.9907  0.7810  0.9998  0.9997  0.9250  0.9881  0.7618  0.9919 |

**Additional file 1: Table S5.** Results of generalised linear model to investigate the spatiotemporal similarity of within-individual petrel migrations when split into 30-day periods, using earth mover’s distance (EMD) ‘effort’ values. Minimum adequate model is shown and categorical variable ‘period’ is being compared to reference level of whole migration. Significant effects (p < 0.05) are highlighted in bold.

| Variable | Estimate ± SE | t value | P value |
| --- | --- | --- | --- |
| 30-day period EMD comparisons (R^2^ = 0.067) | | | |
| (Intercept) | 490.85 ± 61.98 | 7.9 | **< 0.001** |
| 30-day period* |  |  |  |
| First period | 226.15 ± 88.06 | 2.6 | **0.01** |
| Second period | 391.87 ± 99.15 | 4.0 | **< 0.001** |
| Third period | 440.96 ± 102.52 | 4.3 | **< 0.001** |
| Fourth period | 319.85 ± 94.27 | 3.4 | **< 0.001** |
| Fifth period | 493.25 ± 106.90 | 4.6 | **< 0.001** |
| Sixth period | 476.44 ± 123.96 | 3.8 | **< 0.001** |
| Seventh period | 546.10 ± 212.03 | 2.6 | **0.01** |
| Difference in departure | 4.21 ± 0.85 | 5.0 | **< 0.001** |

**Additional file 1: Table S6.** Pairwise comparisons between levels of the categorical variable ‘30-day period’ from the generalised linear model examining the spatiotemporal similarity of petrel migrations when split into 30-day periods, using earth mover’s distance (EMD) ‘effort’ values. Significant effects (p < 0.05) are highlighted in bold.

| Contrast | Estimate ± SE | P value |
| --- | --- | --- |
| Whole migration – first 30-day period  Whole migration – second 30-day period  Whole migration – third 30-day period  Whole migration – fourth 30-day period  Whole migration – fifth 30-day period  Whole migration – sixth 30-day period  Whole migration – seventh 30-day period  First 30-day period – second 30-day period  First 30-day period – third 30-day period  First 30-day period – fourth 30-day period  First 30-day period – fifth 30-day period  First 30-day period – sixth 30-day period  First 30-day period – seventh 30-day period  Second 30-day period – third 30-day period  Second 30-day period – fourth 30-day period  Second 30-day period – fifth 30-day period  Second 30-day period – sixth 30-day period  Second 30-day period – seventh 30-day period  Third 30-day period – fourth 30-day period  Third 30-day period – fifth 30-day period  Third 30-day period – sixth 30-day period  Third 30-day period – seventh 30-day period  Fourth 30-day period – fifth 30-day period  Fourth 30-day period – sixth 30-day period  Fourth 30-day period – seventh 30-day period  Fifth 30-day period – sixth 30-day period  Fifth 30-day period – seventh 30-day period  Sixth 30-day period – seventh 30-day period | -226.2 ± 88.1  -391.9 ± 99.1  -441.0 ± 102.5  -319.9 ± 94.3  -493.2 ± 106.9  -476.4 ± 124.0  -546.1 ± 212.0  -165.7 ± 110.0  -214.8 ± 113.0  -93.7 ± 105.6  -267.1 ± 117.0  -250.3 ± 132.9  -320.0 ± 217.5  -49.1 ± 121.8  72.0 ± 115.0  -101.4 ± 125.5  -84.6 ± 140.5  -154.2 ± 222.3  121.1 ± 117.9  -52.3 ± 128.2  -35.5 ± 142.9  -105.1 ± 223.8  -173.4 ± 121.7  -156.6 ± 137.1  -226.3 ± 220.1  16.8 ± 146.1  -52.9 ± 225.8  -69.7 ± 234.1 | 0.1676  **0.0020**  **0.0005**  **0.0159**  **0.0001**  **0.0031**  0.1648  0.8040  0.5502  0.9873  0.3030  0.5625  0.8230  0.9999  0.9985  0.9928  0.9989  0.9972  0.9704  0.9999  1.0000  0.9998  0.8461  0.9475  0.9703  1.0000  1.0000  1.0000 |

**Additional file 1: Table S7.** Colour of geographic coordinates in figures (main text and Additional File 1) and the corresponding year/s of each migration.

| Figure | Colour of geographic coordinates | | | |
| --- | --- | --- | --- | --- |
|  | **Orange** | **Blue** | **Red** | **Green** |
| 2a | 2016/17 | 2017 | 2011 | 2010/11 |
| 2b | 2016/17 | 2017 | 2012 | 2011 |
| 2c | 2015/16 | 2016/17 | 2011 | - |
| 2d | 2011 | 2012 | 2015/16 | - |
| 2e | 2011 | 2011/12 | 2015/16 | - |
| 2f | 2015/16 | 2016 | - | - |
| 2g | 2010 | 2011 | - | - |
| 2h | 2011 | 2012 | - | - |
| 2i | 2011 | 2011/12 | - | - |
| 2j | 2009/10 | 2010/11 | - | - |
| 2k | 2015 | 2016 | - | - |
| 2l | 2015/16 | 2016 | - | - |
| 3a | 2011 | 2011/12 | - | - |
| 3b | 2015/16 | 2016 | - | - |
| 3c | 2016/17 | 2017 | - | - |
| 3d | 2012 | 2011 | - | - |
| 3e | 2012 | 2016 | - | - |
| 3f | 2009/10 | 2009/10 | - | - |
| S3a | 2009/10 | 2009/10 | - | - |
| S3b | 2017 | 2010 | - | - |
| S3c | 2015/16 | 2016/17 | - | - |

**Additional file 1: Table S8.** The variance components and repeatability estimates (*R*) from adult Round Island petrels with repeated tracks (2-5 years) for departure date from the breeding colony, arrival at the colony and duration of the migratory period.

|  | Between-individual variance | Within-individual variance | *R* |
| --- | --- | --- | --- |
| Departure date | 8148.0 | 2203.2 | 0.787 |
| Arrival date | 8669.5 | 1998.7 | 0.813 |
| Duration | 369.76 | 425.62 | 0.465 |

**Additional file 1:**
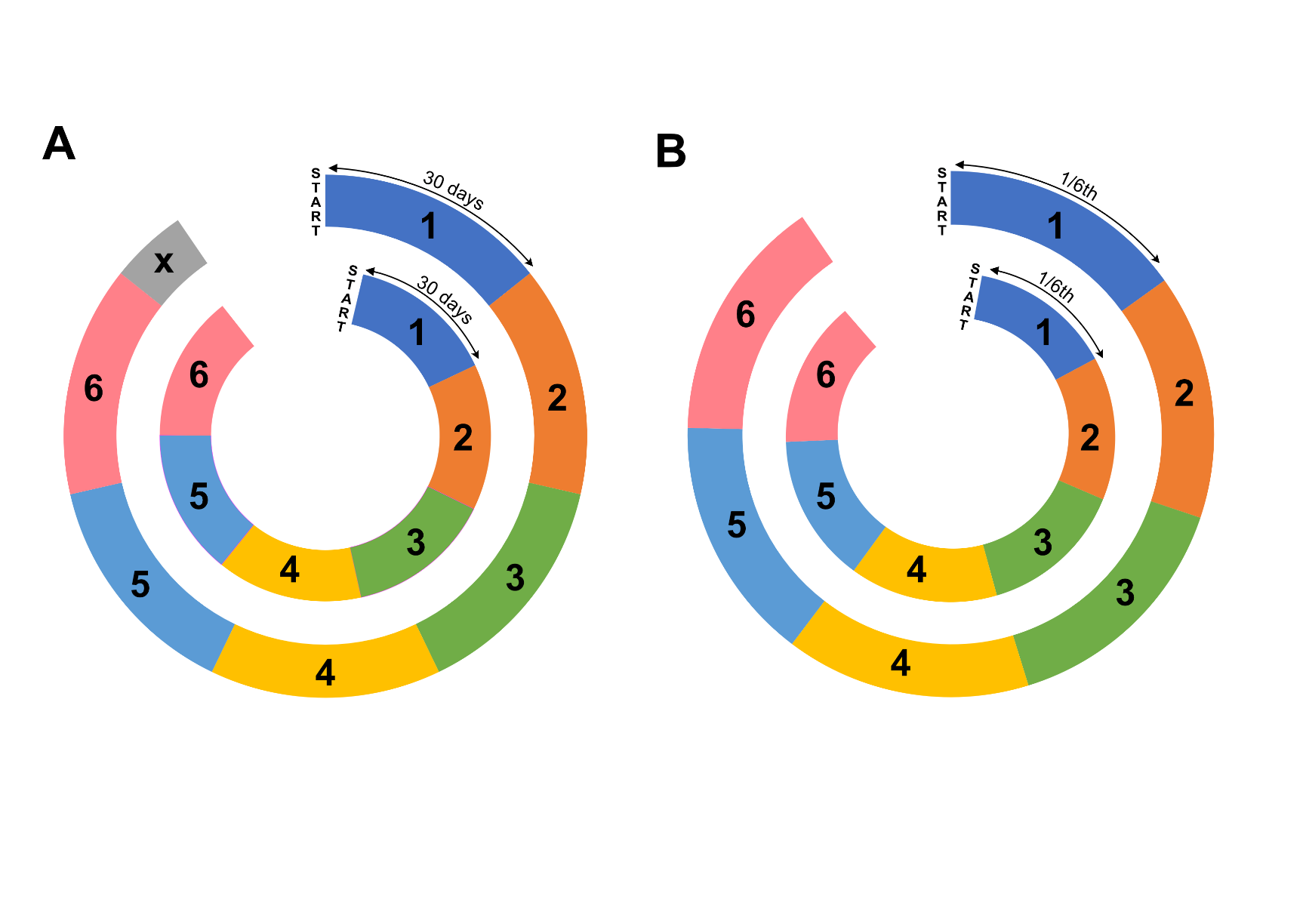
**Figure S1.** Earth mover’s distance (EMD) values are calculated for both A) consecutive 30-day periods, irrespective of start date, and B) by splitting the migration into six equal stages. For A), this typically means that a short period of longer within-individual migrations is not included, however, there was evidence of individual consistency in migration duration and so this often only comprises a small proportion of the whole migration. Whereas for B), if one migration is longer than the other then each stage will comprise of more days than the other corresponding stage.

**Additional file 1:**
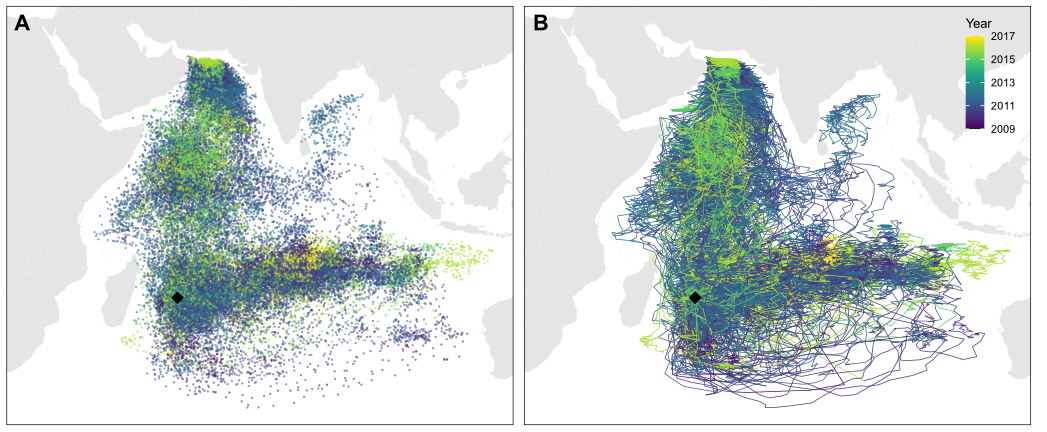
 **Figure S2.** Geographic coordinates (a) and tracks (b) from 62 adult Round Island petrels that have been tracked over multiple complete migrations (n=131) with geolocators. Colours of lines and points represent the year of tracking (2009-17). Black diamond indicates the location of Round Island, Mauritius.

**Additional file 1:**
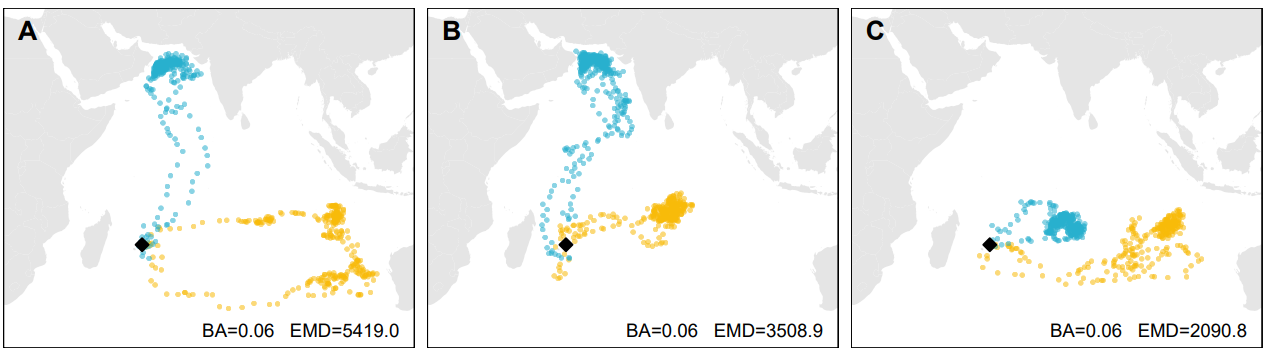
**Figure S3.** Example tracks of between-individual Round Island petrel migration comparisons with the same relative overlap value (Bhattacharyya's affinity (BA) = 0.06), but with comparatively high (A), moderate (B), and low (C) earth mover’s distance ‘effort’ values. Positions denote twice-daily median locations with the two different years illustrated in different colours. Black diamond indicates the location of Round Island, Mauritius. The tracking year that each set of colours represents can be found in Additional File 1: Table S7.
